# Breastfeeding Versus Bottle Feeding and Risk of Bronchial Asthma in Children in Tabuk Area, Saudi Arabia: A Cross-Sectional Study

**DOI:** 10.7759/cureus.68330

**Published:** 2024-08-31

**Authors:** Sawsan Mohammed Alblewi, Muhanned Amawi, Amjad Fiusal H Alharthe, Dana K Alqoaer, Reema Saleh A Albalawi, Lulwah Saud A Alkhuraisi, Reham Hamed A Alrahil, Rahaf Hamed A Alrahil, Rahaf Masoud D Albalawi

**Affiliations:** 1 Pediatrics, Faculty of Medicine, University of Tabuk, Tabuk, SAU; 2 Pediatrics, King Salman Armed Forces Hospital, Tabuk, SAU; 3 Medicine and Surgery, Faculty of Medicine, University of Tabuk, Tabuk, SAU

**Keywords:** bottle feeding, breastfeeding, asthma, children, saudi arabia

## Abstract

Introduction

Asthma is a common chronic airway disorder, particularly among children in Saudi Arabia, with an increasing global prevalence. Limited epidemiological studies in the region indicate variable asthma rates, potentially influenced by factors such as modernization and environmental changes. Given the potential health benefits of breastfeeding, it is critical to investigate the relationship between type of feeding, and asthma risk. This study aims to assess the relationship between breastfeeding and bottle-feeding practices and the risk of developing asthma, as well as to evaluate how breastfeeding practices influence the severity of asthma among children in Tabuk, Saudi Arabia.

Methods

A cross-sectional study was conducted among children with bronchial asthma at King Salman Armed Forces Hospital in Tabuk City, Saudi Arabia. The study took place from June to August 2023 using convenient sampling. Data were collected through a validated, structured web-based questionnaire distributed to the parents of children with bronchial asthma. The analysis was performed using SPSS (IBM SPSS Statistics for Windows, Version 20.0. Armonk, NY: IBM Corp).

Results

The study comprised 103 participants, of whom 66 (64.1%) were breastfed. There was no significant association between the severity of asthma and breastfeeding practices, including the initiation time, duration, or the age at which formula milk was introduced, as well as the combination of breastfeeding with bottle feeding. However, paternal smoking was significantly associated with asthma (p = 0.003), with 78.5% of children with smoking fathers affected compared to 50% of those without smoking fathers.

In the multivariate analysis, having a father employed in the health sector (p = 0.040) and a maternal age over 35 years (p = 0.026) were significantly associated with an increased risk of asthma. Other factors, such as the child’s age, gender, birth order, monthly income, parental education, family size, type of accommodation, and maternal diet during pregnancy, did not demonstrate significant associations with asthma.

Conclusion

Our study underscores the significant role of paternal smoking and specific parental sociodemographic factors, such as having a father employed in the health sector and maternal age over 35 years, in influencing the risk of childhood asthma. While breastfeeding practices, including duration and method, were not significantly associated with asthma severity, understanding these key risk factors can aid in developing targeted interventions for asthma prevention and management

## Introduction

Asthma is a chronic disorder of the airways that is characterized by reversible airflow obstruction and airway inflammation leading to bronchoconstriction, edema, and increased mucous production in the airways [1]. Clinically, asthma is characterized by repeated attacks of wheezing, coughing, and shortness of breath [2]. According to a 2012 Centers for Disease Control and Prevention (CDC) National Surveillance of Asthma report, asthma prevalence among children increased between 2001 and 2010, and it is more prevalent in boys, children under the age of five, black children, and children of Puerto Rican heritage. Additionally, compared to white children, hospitalizations for asthma were 3.6 times higher and emergency room (ER) visits were three times higher in the case of black children [3]. The International Study of Asthma and Allergies in Childhood (ISAAC) estimates that there were 495 asthma-related fatalities in 2017, and 22.8 million suffer from disability because of asthma [4]. One of the most typical childhood chronic diseases is bronchial asthma [5]. Several recent research indicates that asthma prevalence is rising globally [6]. In Saudi Arabia, few epidemiological studies have been carried out to determine the prevalence of childhood asthma, and the outcomes of those studies have been quite inconsistent. Children and teenagers with asthma are more common than previously thought in Saudi Arabia. Bronchial asthma prevalence was observed by Al-Ghamdi et al. [7], to be 19.5% at sea level and 6.9% at higher altitudes in the southern part of Saudi Arabia. According to Al Ghobian et al. [8], the prevalence rates for males and females with doctor-diagnosed asthma were 25.3% for a lifetime wheeze, 18.5% for a wheeze in the last year, and 19.6% for both. According to studies conducted over the previous three decades, the total prevalence of asthma in children ranges from 8 to 25%, according to the Saudi Initiative for Asthma (SINA 2016). Panels from the Saudi Initiative for Asthma stated that this increase is multifaceted, with some of it possibly connected to the community's quick modernization or environmental causes (mostly sandstorms). A self-reported clinical diagnosis of asthma was found to be present in 4.05% of Saudi households, according to data from a national household survey conducted in 2013. The survey estimated the burden of chronic medical diseases, including asthma, among Saudis aged 15 or older [8].

The most prevalent chronic illness in children is asthma, which places a heavy strain on the sufferer, their family, and society. Asthma significantly contributes to pediatric hospitalizations and school absences [9]. A reduced burden on the patient, their family, and society can result from a better comprehension of the natural course of asthma and greater asthma control. The main burden of asthma is a reduced quality of life for the patient and their family as well as substantial societal costs; in developed nations, asthma-related medical expenses account for 1-2% of all medical expenses [9]. Asthma significantly contributes to pediatric hospitalizations and school absences [10]. There's a theory about the relationship between asthma and breastfeeding in children. Indeed, a lot of studies worldwide emphasize that breastfeeding confers several benefits on short- and long-term health which include reducing the risk of many chronic diseases such as food allergies, allergic rhinitis, atopic dermatitis, and asthma but there’s no research evidence in our region about it. Therefore, we aimed to investigate the association between breastfeeding vs bottle feeding and asthma risk among children in Tabuk, Saudi Arabia. This study is based on gathering information from the parents of children with bronchial asthma and detailed information about bronchial asthma and breast and bottle feeding.

Breastfeeding offers numerous advantages, including lowering the risk of many chronic diseases [11]. Data from the United States National Health and Nutrition Examination Survey (NHANES) from 1999 to 2014 were analyzed to examine the association between the duration of exclusive breastfeeding and asthma in 6000 children aged three to six years. The study found that exclusive breastfeeding, especially for four to six months, is associated with a decreased risk of asthma in preschool-aged children. However, this protective effect diminished in older children [11].

A 2017 review summarizing existing evidence on breastfeeding and human milk composition about allergic disease development suggests that allergic diseases, such as eczema, food allergies, and asthma, are the most common chronic diseases of childhood in many countries. Evidence suggests that early life events, such as variations in breastfeeding patterns, maternal diet, and environmental and microbial exposures, may be important in their development [12]. Furthermore, many systematic reviews and meta-analyses have investigated this association. One meta-analysis published in 2021 included retrospective and prospective cohorts of children aged less than 18 years and suggested that the duration and exclusivity of breastfeeding are associated with a lower risk of asthma in children aged less than seven years [13].

Another review investigated infant milk-feeding practices and their relation to food allergies, allergic rhinitis, atopic dermatitis, and asthma throughout the lifespan. The conclusion was that there is moderate evidence suggesting that feeding human milk for short durations or not at all is associated with a higher risk of childhood asthma. Lastly, a review published in 2018 summarized current evidence on this topic and concluded that the impact of breastfeeding on asthma remains unclear, highlighting the need for further epidemiological and biomedical research to establish the association between breastfeeding and asthma development, establish causality, and characterize the underlying biological mechanisms [10].

Consequently, an effective strategy to decrease the incidence of pediatric asthma is needed, particularly focusing on the early postnatal period, including the source of nutrients. This study aims to explore the relationship between breastfeeding and bottle-feeding practices and the risk of developing asthma, while also examining how breastfeeding practices affect the severity of asthma among children in Tabuk, Saudi Arabia.

## Materials and methods

Study design and setting

This cross-sectional study was conducted from June to August 2023 in the Tabuk region of Saudi Arabia. Approval for the research was obtained from the Research Ethics Committee at King Salman Armed Forces Hospital (KSAFH) with the number KSAFH-REC-2023-521. Informed consent was obtained from all participants before their inclusion in the study. Data were collected using a modified questionnaire based on previous studies by Alatawi et al. [14] and Hus et al. [15]. The questionnaire was validated by two pediatric consultants and distributed to parents of children with bronchial asthma at King Salman Armed Forces Hospital, encompassing the pediatric/chest clinic, pediatric emergency room, and pediatric inpatient department. The sample size for the study was determined using the Qualtrics calculator, resulting in a sample of 103 patients. This calculation was based on a total population of 140 patients, with a 95% confidence level and a 5% margin of error [16].

Inclusion and exclusion criteria

The study included children with bronchial asthma who were younger than 14 years old and older than one year. excluding children born preterm with a gestational age of less than 37 weeks, children with a birth weight of less than 2.5 kg, and those with congenital diaphragmatic hernia or congenital heart disease.

Sampling technique and data collection tools

A convenient sampling technique was employed to select the participants. Data were gathered through a questionnaire created by the research team. Participants were informed about the study objectives, methodology, risks, and benefits. Informed consent was obtained before participation.

Data entry and statistical analysis

Data were initially entered into Microsoft Office Excel 2016 for Windows and subsequently transferred to SPSS software (IBM SPSS Statistics for Windows, Version 20.0. Armonk, NY: IBM Corp) for statistical analysis. Comprehensive statistical analyses were conducted on the dataset, encompassing both descriptive and inferential methodologies. Descriptive analysis summarized the demographic characteristics of the participants, including age, gender, and other relevant features, providing an overview of the study population. Inferential analyses, such as the Chi-Square and Fisher’s Exact Test, were employed to examine the association between categorical variables. Binary Logistic Regression was used to identify adjusted predictors of bronchial asthma. Statistical significance was established at a p-value of 0.05 or lower with a 95% Confidence Interval. All statistical analyses were executed using IBM SPSS software (IBM SPSS Statistics for Windows, Version 20.0. Armonk, NY: IBM Corp).

Utilization of Data

The data will serve as a baseline for future planning and intervention. The study aimed to determine if there is a relationship between bronchial asthma and the type of feeding. This data can be used in future research on bronchial asthma in the Tabuk area and across Saudi Arabia.

## Results

In our study on the association between breastfeeding or bottle feeding and the risk of bronchial asthma among children in Tabuk, Saudi Arabia, a total of 103 participants were included (Table 1). There were slightly more males 61 (59.2%) than females 42 (40.8%). The age distribution varied, with the majority of children falling within the one to five years category 36 (35.0%). Most participants were of Saudi nationality 98 (95.1%) and the majority were the elder child in their family 50 (48.5%). Regarding monthly income, a significant portion fell within the range of 5000-10,000 SAR 62 (60.2%). Fathers' educational attainment varied, with 52 (50.5%) having a high school education or below and 51 (49.5%) having a college education or higher. Similarly, 51 (49.5%) of mothers had a high school education or below, and 52 (50.5%) had a college education or higher. Family size showed that the majority 67 (65.0%) had three or more children. Accommodation types primarily consisted of four to five-room apartments 57 (55.3%). Most fathers 84 (81.6%) and mothers 95 (92.2%) did not work in the health sector. Additionally, a majority of mothers 67 (65.0%) were under 35 years old at the time of their child's birth.

**Table 1:** Sociodemographic and other parameters of participants

		Frequency (n=103)	Percent
Gender	Female	42	40.8
	Male	61	59.2
Age	1-5 Years	36	35.0
	5-14 Years	67	65.0
Nationality	Non-Saudi	5	4.9
	Saudi	98	95.1
Birth Order of Child	1st Child	21	20.4
	2nd Child	32	31.1
	Elder Child	50	48.5
Monthly Income	<5000 SAR	11	10.7
	5000-10,000 SAR	62	60.2
	>10,000 SAR	30	29.1
Father's level of education	High School or Below	52	50.5
	College Degree	12	11.7
	University Education	39	37.9
Mother’s level of education	High School or Below	51	49.5
	College Degree	45	43.7
	University Education	7	6.8
Number of Children in Family	1	10	9.7
	2	26	25.2
	3 or More	67	65.0
Accommodation Type	1-3 Rooms Apartment	15	14.6
	4-5 Rooms Apartment	57	55.3
	6-7 Rooms Apartment	31	30.1
Is the father's profession related to the health sector?	No	84	81.6
	Yes	19	18.4
Is the mother’s profession related to the health sector?	No	95	92.2
	Yes	8	7.8
Mother's age at birth?	< 35 year	67	65.0
	> 35 year	36	35.0

Table 2 shows various pregnancy, breastfeeding, and family history factors related to asthma among the participants. During pregnancy, most mothers reported eating little or no fish 84 (81.6%) rather than plenty of fish or omega-3 fatty acids 19 (18.4%). Smoking during pregnancy was rare, with only 2 (1.9%) of mothers reporting smoking. Most births were normal deliveries 63 (61.2%), while 40 (38.8%) were via cesarean section. Concerning breastfeeding, the majority of babies started breastfeeding within the first three months 55 (53.4%), and the mean duration of breastfeeding was 11.4 months (SD = 8.4). Additionally, all breastfed infants feed directly from the breast. A family history of asthma or allergic diseases was prevalent in 55 (53.4%), with asthma being the most common condition reported in 15 (14.6%). Furthermore, a notable percentage of participants had family members allergic to animals 25(24.3%), particularly cats/birds 13 (12.6%). Smoking was more prevalent among fathers 65 (63.1%) than mothers 4 (3.9%).

**Table 2:** Different features of pregnancy, breastfeeding, and family history related to Asthma

		Frequency (n=103)	Percent
Mother's Diet during Pregnancy	Eat Little or No Fish	84	81.6
	Plenty of Fish or Omega-3 FA	19	18.4
During pregnancy, did the mother smoke? (cigarette per day)	No	101	98.1
	Yes	2	1.9
Type of Birth?	Normal Delivery	63	61.2
	Cesarean Section	40	38.8
When does the baby start breastfeeding?	Never	37	35.9
	< 3 Months	55	53.4
	> 3 Months	11	10.7
Duration of Breastfeeding?	Never	37	35.9
	For 3 Months	25	24.3
	For 6 Months	14	13.6
	12-24 Months	27	26.2
Breastfeeding Method?	Expressed breast milk from Bottle	0	0
	Milk from Breast Directly	66	100
Age of introducing formula milk for the first time	Never	13	12.6
	< 3 Months	63	61.2
	3-6 Months	15	14.6
	>6 Months	12	11.6
Breastfeeding with formula Milk	Never	50	48.5
	< 3 Months	28	27.2
	3-6 Months	15	14.6
	>6 Months	10	9.7
Family history of asthma or other Allergic Diseases	No	48	46.6
	Yes	55	53.4
If your answer is yes, mention it	Asthma	15	14.6
	Dust Allergy	8	7.8
	Food Allergy	12	11.7
	Others	20	19.4
Are you allergic to Animals?	No	78	75.7
	Yes	25	24.3
If your answer is yes, mention it	Cats/Birds	13	12.6
	All Animals	8	7.7
	Rabbits	4	3.8
Is the father a smoker?	No	38	36.9
	Yes	65	63.1
Is the mother a smoker?	No	99	96.1
	Yes	4	3.9

Table 3 shows the prevalence and various features of bronchial asthma among the children included in the study. A significant portion of children were not dependent on fast food 66 (64.1%). Most households experienced trucks passing near their houses scarcely 77 (74.8%). Concerning asthma history, all patients had wheezing or wheezing in the chest in the past 12 months 103 (100 %). The majority reported one to three episodes 69 (67.0%). Additionally, wheezing during or after exercise was reported by half of the children 52 (50.5%), and a considerable percentage had a dry cough at night in the past 12 months 63 (61.2%). Furthermore, a substantial portion experienced wheezing episodes severe enough to affect their breathing while speaking 37 (35.9%).

**Table 3:** Prevalence and different features of bronchial asthma among children

		Frequency (n=103)	Percent
Does the child dependent on fast food?	No	66	64.1
	Yes	37	35.9
Do trucks pass near the house?	Scarcely	77	74.8
	Alot	26	25.2
Has the child had wheezing or wheezing in the chest in the past 12 months?	No	0	0
	Yes	103	100
If yes, how many wheezing episodes has your child experienced?	1-3	69	67.0
	4-12	26	25.2
	>12	8	7.8
In the past 12 months, has the child's chest been wheezing during or after exercise?	No	51	49.5
	Yes	52	50.5
In the past 12 months, has the child had a dry cough at night, other than a cough associated with a cold or chest infection?	No	40	38.8
	Yes	63	61.2
In the last 12 months, on average how many times has your child had to wake up due to wheezing?	Never woke up	35	34.0
	Less than One Night a Week	37	35.9
	One or More Nights Week	31	30.1
In the last 12 months, has your child had any wheezing or wheezing in the chest so severe that it affected his breathing while speaking?	No	66	64.1
	Yes	37	35.9

Table 4 presents the baby's feeding profile. As for the type of feeding, 66 (64.1%) reported that the child received breastfeeding, while 37 (35.9%) reported that the child did not receive breastfeeding and only received formula feeding. Among those who received breastfeeding, 61 (94.42%) reported that the child started breastfeeding at an age younger than three months, while 5 (7.58%) reported that the child received breastfeeding between the ages of three to six months. As for the duration of breastfeeding, 25 (37.88%) reported it was for three months, 15 (22.73%) reported it was for six months, 9 (13.64%) reported it was for 12 months, 7 (10.61%) reported it was for 18 months, and 10 (15.15%) reported it was for 24 months. As for the age of first introducing milk formula, 12 (11.7%) reported they never did, 65 (63.1%) reported it was at an age younger than three months, 15 (14.6%) reported it was between three to six months, and 11 (10.7%) reported it was at an age older than six months. As for combining breastfeeding and formula feeding, 50 (48.5%) reported they never did it, 30 (29.1%) reported they did it at an age younger than three months, 13 (12.6%) reported they did it at an age between three to six months, and 10 (9.7%) reported they did it at an age older than six months.

**Table 4:** Baby feeding profile

Question	n	%
Breastfeeding practice		
Received breastfeeding	66	64.1
Did not receive breastfeeding, only formula feeding	37	35.9
Starting of breastfeeding (n = 66)		
Younger than 3 months	61	92.42
3 - 6 months	5	7.58
Duration of breastfeeding (n = 66)		
For 3 months	25	37.88
For 6 months	15	22.73
For 12 months	9	13.64
For 18 months	7	10.61
For 24 months	10	15.15
The age of first introducing milk formula		
Never	12	11.7
Younger than 3 months	65	63.10
3 - 6 months	15	14.60
Older than 6 months	11	10.70
Breastfeeding combined with bottle formula feeding		
Never	50	48.5
Younger than 3 months	30	29.1
3 - 6 months	13	12.6
Older than 6 months	10	9.7

Table 5 shows the factors associated with severity of asthma. The severity of asthma was not significantly associated with breastfeeding practice, time of starting breastfeeding, duration of breastfeeding, age at first introducing formula milk, and giving breastfeeding combined with bottle formula feeding.

**Table 5:** Factors associated with severity of asthma

Factor	Severity of Asthma		P-Value
	Mild	Moderate to Severe	
Breastfeeding practice			
Received breastfeeding	42 (63.6%)	24 (36.4%)	0.334
Did not receive breastfeeding, only formula feeding	27 (73%)	10 (27%)	
Starting of breastfeeding			
Younger than 3 months	38 (62.3%)	23 (37.7%)	0.429
3 - 6 months	4 (80%)	1 (20%)	
Duration of breastfeeding			
For 3 months	18 (72%)	7 (28%)	
For 6 months	10 (66.7%)	5 (33.3%)	0.328
For 12 months	3 (33.3%)	6 (66.7%)	
For 18 months	5 (71.4%)	2 (28.6%)	
For 24 months	6 (60%)	4 (40%)	
The age of first introducing milk formula			
Younger than 3 months	47 (72.3%)	18 (27.7%)	0.667
3 - 6 months	8 (53.3%)	7 (46.7%)	
Older than 6 months	7 (63.6%)	4 (36.4%)	
Breastfeeding combined with bottle formula feed			
Never	34 (68%)	16 (32%)	
Younger than 3 months	22 (73.3%)	8 (26.7%)	0.614
3 - 6 months	7 (53.8%)	6 (46.2%)	
Older than 6 months	6 (60%)	4 (40%)	
*Significant at level 0.05			

Table 6 shows the outcomes of a multivariate analysis investigating sociodemographic predictors of bronchial asthma among children. Among the examined factors, significant associations were observed for the father's profession related to the health sector and the mother's age at childbirth. Children whose fathers worked in health-related professions were substantially more likely to have asthma [p=0.040, Exp(B) = 6.924], indicating a heightened risk compared to those whose fathers were not in such professions. Similarly, children born to mothers over 35 years old exhibited a significantly increased likelihood of developing asthma [p=0.026, Exp(B) = 3.562], emphasizing a threefold higher risk compared to those born to younger mothers. Other factors, including the child's age, gender, birth order, monthly income, parental education level, number of children in the family, type of accommodation, and mother's diet during pregnancy, did not show statistically significant associations with bronchial asthma.

**Table 6:** Sociodemographic predictors of bronchial asthma among child (multivariate analysis)

	B	Sig.	Exp(B)	95% CI	
				Lower	Upper
Child age	.159	.526	1.172	.718	1.913
Gender (male)	-.343	.531	.710	.243	2.075
Birth order of child	-.111	.785	.895	.403	1.987
Monthly income	.539	.290	1.714	.632	4.644
Higher education of father	-.612	.065	.542	.283	1.039
Higher education of mother	.144	.765	1.154	.451	2.957
Number of children in family	-.760	.145	.468	.168	1.299
Accommodation (higher rooms)	-.146	.742	.864	.364	2.054
Father work related to health profession	1.935	.040	6.924	1.092	43.884
Mother work related to health profession	20.532	.999	-	-	-
Mother's age at childbirth (>35 Years)	1.270	.026	3.562	1.167	10.870
Mother's diet during pregnancy (Fish/Omega-3 FA)	-.058	.932	.944	.251	3.551
Constant	.960	.600	2.613		

## Discussion

Asthma is a chronic airway disorder, especially among children in Saudi Arabia, with increasing global prevalence. Alahmadi et al. [6], demonstrated the global significance of asthma, highlighting the limited and inconsistent research on its prevalence among Saudi children, which underscores the need for more comprehensive studies. Alomary et al. [17], identified bronchial asthma as one of the common chronic diseases of childhood. Investigating the association between breastfeeding, bottle feeding, and asthma risk is crucial, given the potential health benefits of breastfeeding. Our study aimed to address this gap by gathering data from parents of children with bronchial asthma in Tabuk, Saudi Arabia.

Our study reveals a slightly higher proportion of males (59.2%) compared to females (40.8%), a gender distribution consistent with broader trends in asthma prevalence, which typically shows higher rates among males during childhood but tends to reverse in adulthood. Almqvist et al. [18], reported that the female-to-male prevalence ratio for asthma in children is approximately 35:65. Moreover, asthma tends to be more severe in females than in males after childhood and is often underdiagnosed and undertreated in female adolescents [17]. The majority of children in our study fell within the one to five years age category, reflecting the early onset of asthma symptoms. Lam et al. [19], found that the median onset age of asthma symptoms was three years, peaking between one to three years, with over 90% experiencing symptoms before age six.

Most participants were of Saudi nationality (95.1%), indicating the study's relevance to the local population. The distribution of parental education levels, family size, and accommodation types provides insights into the socioeconomic background of the participants. Additionally, the prevalence of parental smoking and family history of asthma or allergic diseases underscores the multifactorial nature of asthma etiology.

By addressing these factors, our study contributes valuable data to the understanding of asthma risk factors in Saudi children, particularly concerning feeding practices and sociodemographic variables.

Pregnancy-related factors, including maternal diet, smoking status, and mode of delivery, were explored in relation to bronchial asthma among children. The majority of mothers reported little or no fish consumption during pregnancy, reflecting regional dietary habits. Neutze et al. [20], found that omega-3 fatty acid consumption during pregnancy decreased the risk of atopy and asthma in offspring. Similarly, Jia et al. [21], reported that perinatal supplementation with n-3 LC-PUFA reduced the incidence of asthma/wheeze and allergic asthma in children, with higher doses providing better protective effects. Maternal smoking during pregnancy was rare, indicating increasing awareness of the harmful effects of tobacco smoke on fetal health. Miyake et al. [22], highlighted that maternal smoking throughout pregnancy and smoking exposure pre-pregnancy or in early pregnancy increases the risk of bronchial asthma in children. Despite this, the prevalence of asthma among children in our study was notably high, with a significant proportion experiencing wheezing or chest symptoms in the past 12 months. In contrast, Triasih et al. [23], reported a low prevalence of current wheeze in children and adolescents in Indonesia. A family history of asthma or allergies was prevalent in our study, indicating a potential genetic predisposition. Jindal et al. [24], noted that about half of asthma patients have a genetic susceptibility, with a family history of asthma and other atopies providing strong evidence for a genetic basis of asthma.

Our study investigated the association between breastfeeding practices and the risk of bronchial asthma among children. While our findings did not show a significant association between the timing, duration, or method of breastfeeding and asthma risk, other studies have reported different results. For example, Wilson et al. [25] provided evidence for protective associations between longer durations of exclusive breastfeeding (≥2 months) and child asthma outcomes, highlighting the potential benefits of prolonged breastfeeding. Similarly, Hsu et al. [15] found that the use of feeding bottles was associated with an increased risk of wheezing/asthma, suggesting that suboptimal breastfeeding practices might impact respiratory health. In our study, paternal smoking status emerged as a significant predictor of asthma, with children of smoking fathers showing a higher likelihood of asthma compared to those with non-smoking fathers. This finding aligns with the work of Strachan et al. [26], who reported a pooled odds ratio for asthma prevalence of 1.37 if either parent smoked and noted that parental smoking was more strongly associated with wheezing among non-atopic children. Additionally, our study identified other significant sociodemographic factors, such as having a father employed in the health sector and maternal age over 35, as influencing the risk of childhood asthma.

Thus, our study findings align with prior research, underscoring the protective effect of breastfeeding on asthma. Longer breastfeeding duration correlates with a lower risk of asthma. Paternal smoking and maternal age at childbirth also emerged as significant predictors of childhood asthma.

Despite its valuable contributions, this study has several limitations. The cross-sectional design limits the ability to draw causal inferences, and the relatively small sample size may affect the generalizability of the findings. Additionally, the reliance on self-reported data introduces potential recall bias and misclassification errors. Future research employing longitudinal designs and objective measures of asthma diagnosis is recommended to provide more robust insights into the relationship between breastfeeding practices and asthma risk. Moreover, investigating gene-environment interactions and epigenetic mechanisms underlying asthma development could further elucidate the complex etiology of this condition.

## Conclusions

This study provides valuable insights into the association between breastfeeding practices, sociodemographic factors, and bronchial asthma among children in Tabuk, Saudi Arabia. The findings highlight the significance of addressing modifiable risk factors, such as paternal smoking, to mitigate the burden of asthma in childhood. Although our study did not find a direct link between breastfeeding practices and asthma risk, the results contribute to a broader understanding of the complex interplay between environmental and genetic factors in asthma etiology. This study adds to the literature on pediatric respiratory health and informs public health interventions aimed at reducing asthma prevalence and morbidity.
